# Protective Effects of Berberine on Isoproterenol-Induced Acute Myocardial Ischemia in Rats through Regulating HMGB1-TLR4 Axis

**DOI:** 10.1155/2014/849783

**Published:** 2014-11-13

**Authors:** Tianzhu Zhang, Shihai Yang, Juan Du

**Affiliations:** ^1^Changchun University of Chinese Medicine, Changchun 130117, China; ^2^Jilin Agricultural University, Changchun 130118, China; ^3^School of Life Science, Peking University, Beijing 100871, China

## Abstract

Berberine, an isoquinoline alkaloid originally isolated from the Chinese herb *Coptis chinensis* (Huanglian), has been shown to display a wide array of pharmacological activities. The present study was to investigate the effects of berberine against myocardial ischemia produced in rats by isoproterenol. 50 male Sprague-Dawley rats were randomized equally into five groups: a control group, an untreated model group, berberine (30, 60 mg/kg) treatment, or propranolol (30 mg/kg). Rats were treated for 12 days and then given isoproterenol, 85 mg/kg for 2 consecutive days by subcutaneous injection. ST-segment elevation was measured after the last administration. Serum levels of creatine kinase isoenzyme (CK-MB), lactate dehydrogenase (LDH), tumor necrosis factor-*α* (TNF-*α*), and interleukin-6 (IL-6) were measured after the rats were sacrificed. The hearts were excised for determining heart weight index, microscopic examination, high mobility group box 1 (HMGB1), toll-like receptor (TLR4), prodeath protein (Bax), antideath protein (Bcl-2), and tumor necrosis factor (TNF-*α*) protein were determined by western blot. Berberine decreased the ST elevation induced by acute myocardial ischemia, and decreased serum levels of CK-MB, LDH, TNF-*α*, and IL-6. Berberine increased total superoxide dismutase (T-SOD) activity and decreased malondialdehyde (MDA) content in myocardial tissue. Berberine can regulate HMGB1-TLR4 axis to protect myocardial ischemia.

## 1. Introduction

Ischemic heart disease (IHD) is the leading cause of morbidity and mortality in the Western world; and, according to the World Health Organization, it will be the leading cause of death in the world [1, 2]. Despite advances in basic research and clinical improvements, there have been no fundamental breakthroughs in drug treatment.

Isoproterenol, *α* *β*-adrenergic agonist, is known to produce cardiac ischemia due to free radical production by autooxidation [3]. Isoproterenol-induced cardiac ischemia results in increased cardiac enzymes and oxidative stress, abnormal electrocardiograph, and cardiac functions [4].

High mobility group box 1 (HMGB1), a 30 kDa nuclear protein involved in the structural organization of deoxyribonucleic acid (DNA), serves as a mediator of inflammation after being released by necrotic cells or upon cellular activation in various pathological conditions including diverse cardiovascular diseases and myocardial ischemia-reperfusion (I/R) injury [5–8]. Meanwhile, toll-like receptor-4 (TLR4), an important receptor for HMGB1, is also involved in the induction of the inflammatory response and can attenuate the inflammation interruption of the ligands-TLR4 axis via multiple approaches [9, 10].

Berberine, an isoquinoline alkaloid originally isolated from the Chinese herb* Coptis chinensis* (Huanglian), has been shown to display a wide array of pharmacological activities which include antimicrobial, antidiarrhoeal, antidiabetic, antihyperlipidaemic, anti-inflammatory, and antiproliferative effects [11]. In addition, both animal and clinical studies have shown that berberine is beneficial in combating cardiovascular disease [12]. The present study was designed to evaluate the effect of berberine pretreatment on the isoproterenol-induced myocardial damage in a rat model.

## 2. Material and Methods

### 2.1. Materials

This study was approved by the Laboratory Animal Center of the Academy of the China Pharmaceutical University. 50 male healthy Sprague-Dawley rats weighing from 200 to 220 g were purchased from the Animal Experimental Animal Center of Jilin University. Animal experiment was carried out in accordance with the Guidelines for Animal Experiment of Jilin University. The animals were maintained in a temperature controlled room at 20.1–23.1°C and 40–50% humidity, a 12 h light/dark cycle, and free access to water. Berberine with purity of over 98% was purchased from National Institutes for Food and Drug Control (Beijing, China). Isoproterenol was purchased from Shanghai Hefeng Pharmaceutical Co. Ltd. (Shanghai, China). Sodium pentobarbital was purchased from Merck (Germany). Propranolol was purchased from Xi'an Li Jun Pharmaceutical Co. ltd. (Xi'an, China). CK-MB, LDH, T-SOD, MDA, TNF-*α*, and IL-6 test kits were all purchased from Nanjing Jian Cheng Biological Engineering Research Institute (Nanjing City, China): anti-HMGB1 antibody (Cell Signaling Technology, #3935), anti-TLR4 antibody (Cell Signaling Technology, #2246), anti-TNF-*α* antibody (Cell Signaling Technology, #6945), anti-Bax antibody (Cell Signaling Technology, #2272), anti-Bcl-2 antibody (Cell Signaling Technology, #2876), and anti-GAPDH antibody (Cell Signaling Technology, #5174).

### 2.2. Experimental Protocol

The rats were randomly assigned to five groups of 10 rats each: (1) control; (2) isoproterenol; (3) isoproterenol + propranolol (30 mg/kg, orally); (4) isoproterenol + berberine (30 mg/kg, orally); (5) isoproterenol + berberine (60 mg/kg, orally). Rats were pretreated for 14 days and then intoxicated with isoproterenol (isoproterenol, 85 mg/kg except for the control group) by subcutaneous injection on two consecutive days. Blood (3 mL) was collected from the abdominal aorta for serum enzyme assays. After treatment, hearts were excised, rinsed in ice-cold isotonic saline, blotted with filter paper, and homogenized in 0.05 M ice-cold phosphate buffer (pH 7.4) for biochemical assays.

### 2.3. Determination of ST-Segment Elevation and Heart Rate

Electrocardiograms (ECG) recorded ST-segment elevation and heart rate at 20 min after the final injection of isoproterenol or other drugs. ECGs were recorded under pentobarbital sodium anesthesia (22.5 mg/kg) using needle electrodes and a BL-420S Biological Function Experiment System purchased from Chengdu Thaimeng Technology Co. Ltd, (Chengdu, China).

### 2.4. Determination of Heart Weight Index

After rats were sacrificed, the heart tissues were excised (excluding large blood vessels and connective tissue) and weighed after blotting with filter paper. The heart weight index (HWI) was computed as HWI = heart weight (HW)/bodyweight (BW).

### 2.5. Determination of CK-MB, LDH, TNF-*α*, and IL-6 in the Serum

CK-MB and LDH levels were measured by a rate assay using an RT-9600 Semiautomatic Biochemical Analyzer (ShenZhenLeiDu Life Science, LLC). TNF-*α* and IL-6 levels were measured by enzyme linked immunosorbent assay (Wuhan Boshide Biological Technology Company, Wuhan, China). All measurements were performed according to the kit manufacturers' instructions.

### 2.6. Histological Examination of Myocardium

Immediately after the sacrifice of the rats, the hearts were removed and fixed in 10% formalin solution. The heart tissue was processed for sectioning and staining by standard histological methods. Sections (5 mm, Leica RM 2125, Germany) from the left ventricle were stained with hematoxylin and eosin (H&E) and examined by light microscopy (Nikon, Tokyo, Japan) at 200x magnification.

### 2.7. Western Blotting

The heart tissues were homogenized, washed with PBS, and incubated in lysis buffer in addition to a protease inhibitor cocktail (Sigma, St. Louis, MO) to obtain extracts of lung proteins. The samples were loaded to 10% SDS-PAGE gels and were electrotransferred to nitrocellulose. The blots were incubated with the appropriate concentration of specific antibody. After washing, the blots were incubated with horseradish peroxidase-conjugated second antibody. The membranes were stripped and reblotted with anti-*β*-actin antibody (Sigma) to verify the equal loading of protein in each lane. Quantification of protein expression was normalized to *β*-actin using a densitometer (Imaging System).

### 2.8. Statistical Analysis

All values were expressed as the mean ± S.D. and analyzed by one-way analysis of variance (ANOVA) followed by Duncan's Multiple Range Test using SPSS version 13.0 software; a *P* value of less than 0.05 was considered significant and *P* < 0.01 was considered to be statistically very significant.

## 3. Results

### 3.1. Effect of Berberine on ST-Segment Elevation

**Figure 1:**
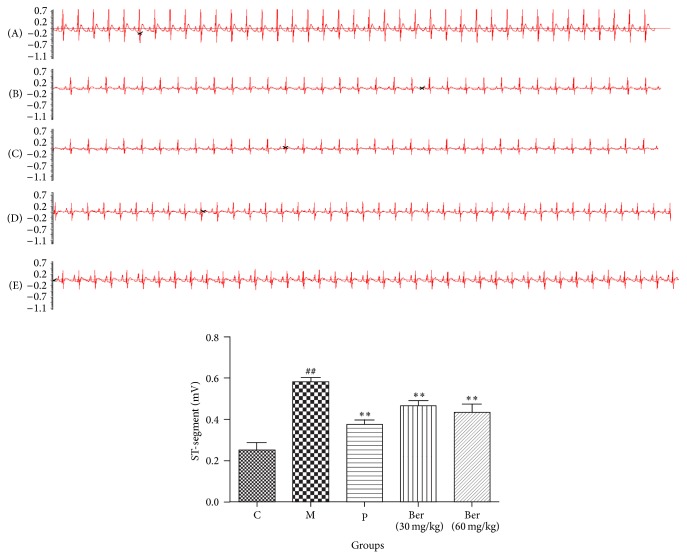
Effect of berberine on ST-segment elevation. C (control); M (model); P (propranolol (30 mg/kg)); D (berberine (30 mg/kg)); E (berberine (60 mg/kg)). Values are expressed as means ± SDs. Compared with control, ^#^ *P* < 0.05, ^##^ *P* < 0.01; compared with model, ^∗^ *P* < 0.05, ^∗∗^ *P* < 0.01.

Five minutes after isoproterenol administration, the ST-segment was elevated in the untreated model group but not in the groups treated with berberine. These results represented that the myocardial ischemia damage model has been established. Ten minutes after isoproterenol administration, ST-segment elevation was still seen in the untreated group. ST-segment elevation was reduced in the berberine groups compared with the untreated model rats. Heart rates tended to stabilize and approximate the rate observed in the propranolol treated group (Figure 1).

### 3.2. Effects of Berberine on CK-MB, LDH, TNF-*α*, and IL-6 Serum Levels

**Figure 2:**
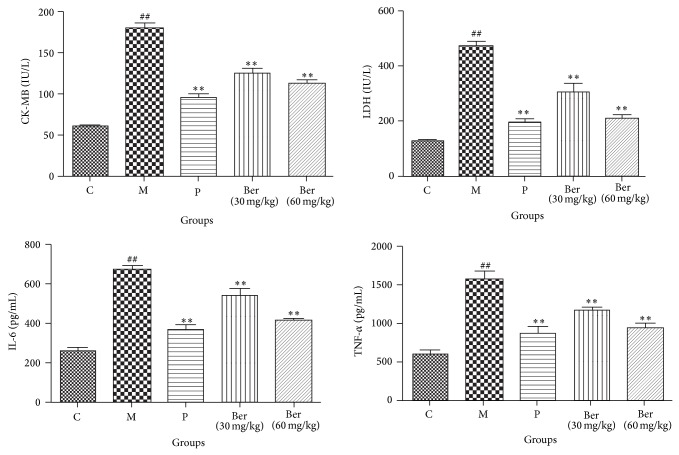
Effects of berberine on CK-MB, LDH, TNF-*α*, and IL-6 serum levels. C (control); M (model); P (propranolol (30 mg/kg)); D (berberine (30 mg/kg)); E (berberine (60 mg/kg)). Values are expressed as means ± SDs. Compared with control, ^#^ *P* < 0.05, ^##^ *P* < 0.01; compared with model, ^∗^ *P* < 0.05, ^∗∗^ *P* < 0.01.

Significant increases in the myocardial injury marker enzymes, CK-MB, and LDH were observed in the untreated model rats compared with the control rats. Pretreatment with berberine decreased CK-MB and LDH levels compared with rats in the untreated model group in a dose-dependent manner. Compared with the control group, serum TNF-*α* and IL-6 levels increased significantly in the untreated model group. Pretreatment with berberine decreased serum TNF-*α* and IL-6 levels compared with the untreated model group rats in a dose-dependent manner (Figure 2).

### 3.3. Effect of Berberine on Heart Weight Indices

**Figure 3:**
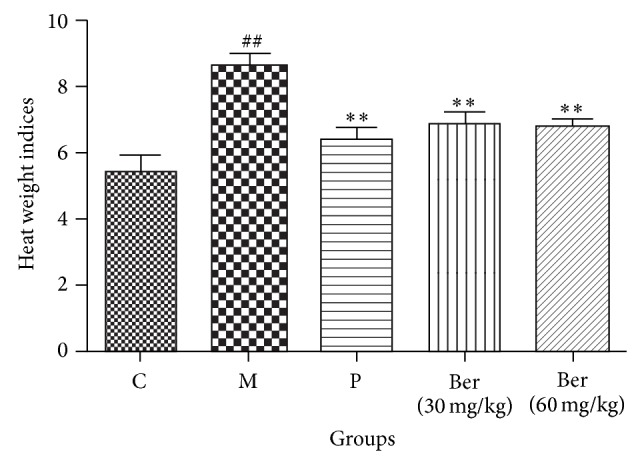
Effect of berberine on heart weight indices. C (control); M (model); P (propranolol (30 mg/kg)); D (berberine (30 mg/kg)); E (berberine (60 mg/kg)). Values are expressed as means ± SDs. Compared with control, ^#^ *P* < 0.05, ^##^ *P* < 0.01; compared with model, ^∗^ *P* < 0.05, ^∗∗^ *P* < 0.01.

HWI were greater in the untreated model rats than in the control group rats. Pretreatment with berberine decreased the HWI compared with the untreated model group rats (Figure 3). As the dose increased, the decrease in HWI became larger.

### 3.4. Effect of Berberine on Myocardial Histology

**Figure 4:**
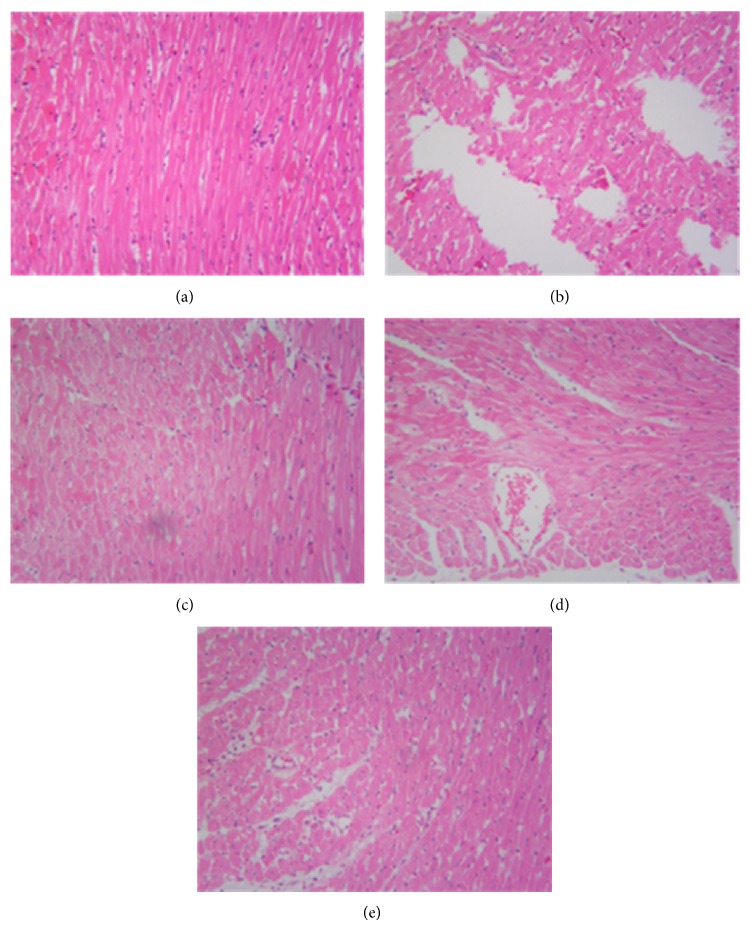
Effect of berberine on myocardial histology (×200). C (control); M (model); P (propranolol (30 mg/kg)); D (berberine (30 mg/kg)); E (berberine (60 mg/kg)).

Light microscopy of tissue sections from control rat myocardium showed a normal myofibrillar structure with striations, branched appearance, and continuity with adjacent myofibrils. Tissue from the untreated model rats given isoproterenol revealed obvious myocardial cell swelling, degeneration, loss of transverse striations, and large numbers of infiltrating inflammatory cells. Tissues from rats pretreated with berberine showed normal, well preserved of cardiac muscle cell histology. Tissue sections from the propranolol group rats revealed approximately normal myofibrillar structure with clear transverse striations and presence of a few inflammatory cells (Figure 4).

### 3.5. Effect of Berberine on HMGB1-TLR4 Axis

**Figure 5:**
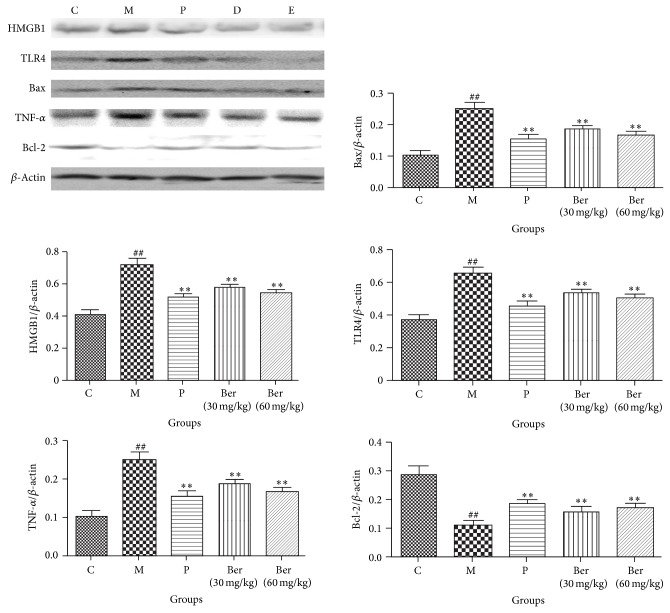
Effect of berberine on HMGB1-TLR4 axis signaling C (control); M (model); P (propranolol (30 mg/kg)); D (berberine (30 mg/kg)); E (berberine (60 mg/kg)). Values are expressed as means ± SDs. Compared with control, ^#^ *P* < 0.05, ^##^ *P* < 0.01; compared with model, ^∗^ *P* < 0.05, ^∗∗^ *P* < 0.01.

The expressions of proteins of HMGB1, TLR4, Bax, Bcl-2, and TNF-*α* were changed by isoproterenol in heart. As shown in Figure 5, compared with the control group, the protein levels of HMGB1, TLR4, Bax, and TNF-*α* in model group were significantly increased. In berberine, the protein levels of HMGB1, TLR4, Bax, and TNF-*α* were significantly decreased in a dose-dependent manner compared to the model group, respectively; compared with the control group, the protein levels of Bcl-2 in model group were significantly decreased. In berberine, the protein levels of Bcl-2 were significantly increased in a dose-dependent manner compared to the model group, respectively.

## 4. Discussion

In this study, berberine reduced the ST-segment elevation induced by acute myocardial ischemia; alleviated myocardial ischemic injury; decreased HWI. Pretreatment with berberine also decreased CK-MB, LDH, TNF-*α*, and IL-6 levels. Berberine pretreatment increased SOD activity and decreased MDA levels in the myocardium. These results suggested that berberine in the doses used in this study had cardioprotective effects in myocardial ischemia that could be attributed to their antioxidative and anti-inflammatory properties.

The acute myocardial ischemia induced by isoproterenol was confirmed by loss of integrity of myocardial membranes on histological examination, ST-segment elevation, and serum elevation of CK-MB and LDH enzymes. Berberine alleviated myocardial histological injury, reduced heart rate and ST-segment elevation, and decreased CK-MB and LDH enzyme in a dose-dependent fashion.

Isoproterenol is a synthetic *β*-adrenergic agonist that can cause severe stress in the myocardium and necrosis of heart muscle cells. However there is a substantial literature [1, 13] demonstrating that estrogen can protect the heart from ischemic injury and inhibit arteriosclerosis and myocardial hypertrophy; that means that estrogen would have affected the experimental results if we use the female rats. So in the current study, we only used the male rats to build the acute myocardial ischemia induced by isoproterenol to contain the study. The acute myocardial ischemia induced by isoproterenol was confirmed by loss of integrity of myocardial membranes on histological examination, ST-segment elevation, rapid heart rate, and serum elevation of CK-MB and LDH enzymes; berberine alleviated myocardial histological injury, reduced heart rate and ST-segment elevation, and decreased CK-MB and LDH enzyme in a dose-dependent fashion.

As a previous study [14], HE staining is an important method for evaluating myocardial injury. Histopathological examination revealed higher degree of myocardial cell swelling, degeneration, loss of transverse striations, and large numbers of infiltrating inflammatory cells in isoproterenol-induced rats. In the current study, treatment with berberine restored the histopathological change.

Inflammation has been recognized as a major driving force in the ischemic process, and increasing evidence has shown that enhanced levels of inflammatory markers are related to ischemia [15, 16]. The proinflammatory cytokines such as TNF-*α* and IL-6 are small secreted proteins that mediate and regulate inflammation. The inflammatory stresses induced by isoproterenol were reflected by TNF-*α* and IL-6 elevation and infiltration of the myocardium by neutrophil granulocytes. Berberine decreased TNF-*α* and IL-6 levels in the serum, which suggested that their cardioprotective effects were also associated with anti-inflammatory properties.

The levels of SOD and MDA activity are among the principal pathophysiological parameters in evaluating free radical metabolism. SOD activity reflects the cellular capability of scavenging/quenching free radicals [17]. In this study, SOD activity was significantly decreased in the heart tissue of isoproterenol treated rats as compared to controls. Pretreatment with Rg1 increased SOD activity and decreased MDA level in the myocardium, which suggested that their cardioprotective effects were related to antioxidative properties.

High mobility group box 1 protein (HMGB1), a highly conserved nuclear protein that could regulate gene transcription and maintain the nucleosome structure, could be passively released from necrotic cell or apoptotic cell or actively secreted by innate immune cells (such as macrophages and monocytes) [18]. Present study shows that HMGB1 as a novel proinflammatory cytokine and contributes to the pathophysiological progress of myocardial I/R injury [19]. HMGB1 may promote the release of TNF-*α*, IL-6 which could be inhibited by HMGB1 A box peptide (a specific HMGB1 antagonist) and attenuate myocardial I/R injury, demonstrating that inhibiting HMGB1 expression could suppress the local myocardial inflammation in myocardial I/R injury. Hence, in the current study, isoproterenol could activate HMGB1, and berberine significantly decreased HMGB1 level in isoproterenol-induced rats.

Several toll-like receptors (TLRs), TLR4, play important roles in mediating myocardial inflammatory and injurious responses to I/R [20, 21]. Several studies have identified high mobility group box 1 (HMGB1) and large heat shock protein (HSP) as endogenous TLR4 activators in animal models of myocardial I/R [22, 23]. As expected, in this study, berberine significantly decreased TLR 4 level in isoproterenol-induced rats.

Inflammation is involved in many cardiovascular diseases, such as myocardial infarction, atherosclerosis, and cardiomyopathy [24–26]. In the process of inflammation, TNF-*α* is of great significance. TNF-*α* is secreted mainly by macrophages, which is likely to promote inflammatory cascade by increasing releases of other proinflammatory cytokines and influencing neutrophil recruitment [27]. TNF-*α* an induce the release of other inflammatory mediators, increase the expression of cell adhesion factor, and promote neutrophil adhesion to endothelial cells. TNF-*α* can also induce cardiomyocyte apoptosis and participate in ventricular remodeling [28]. In current study, isoproterenol could increase TNF-*α*; berberine decreased significantly TNF-*α* in isoproterenol-induced rats.

Cardiomyocyte apoptosis is involved in various heart diseases, such as myocardial hypertrophy, heart failure, and myocardial ischemia [29]. Apoptosis is positively and negatively regulated by the Bcl-2 protein family. Proapoptotic proteins include Bax, Bak, Bcl-XS, Bad, Bid, Bik, Bim, Hrk, and Bok, whereas antiapoptotic proteins include Bcl-2, Bcl-XL, Bcl-w, Mcl-1, and A1/Bfl-1. In the current study, isoproterenol decreased Bcl-2 and increased Bax, while berberine restored the Bax/Bcl-2 balance in isoproterenol-induced rats.

In conclusion, this study demonstrated that berberine had cardioprotective effects against acute ischemic myocardial injury induced by isoproterenol in rats.
